# Pyramidal Cells Make Specific Connections onto Smooth (GABAergic) Neurons in Mouse Visual Cortex

**DOI:** 10.1371/journal.pbio.1001932

**Published:** 2014-08-19

**Authors:** Rita Bopp, Nuno Maçarico da Costa, Björn M. Kampa, Kevan A. C. Martin, Morgane M. Roth

**Affiliations:** 1 Institute for Neuroinformatics, University of Zürich and ETH Zürich, Zürich, Switzerland; 2 Brain Research Institute, University of Zürich, Zürich, Switzerland; 3 Institute de Neurosciences de la Timone, Marseille, France; McGill University, Canada

## Abstract

One of the hallmarks of neocortical circuits is the predominance of recurrent excitation between pyramidal neurons, which is balanced by recurrent inhibition from smooth GABAergic neurons. It has been previously described that in layer 2/3 of primary visual cortex (V1) of cat and monkey, pyramidal cells filled with horseradish peroxidase connect approximately in proportion to the spiny (excitatory, 95% and 81%, respectively) and smooth (GABAergic, 5% and 19%, respectively) dendrites found in the neuropil. By contrast, a recent ultrastructural study of V1 in a single mouse found that smooth neurons formed 51% of the targets of the superficial layer pyramidal cells. This suggests that either the neuropil of this particular mouse V1 had a dramatically different composition to that of V1 in cat and monkey, or that smooth neurons were specifically targeted by the pyramidal cells in that mouse. We tested these hypotheses by examining similar cells filled with biocytin in a sample of five mice. We found that the average composition of the neuropil in V1 of these mice was similar to that described for cat and monkey V1, but that the superficial layer pyramidal cells do form proportionately more synapses with smooth dendrites than the equivalent neurons in cat or monkey. These distributions may underlie the distinct differences in functional architecture of V1 between rodent and higher mammals.

## Abstract 2

Light and electron microscopy of the primary visual cortex of mice indicates that pyramidal neurons connect preferentially to inhibitory neurons.

## Author Summary

The mammalian visual cortex, which is part of the cerebral cortex, contains 50 to 100 thousands of neurons per cubic millimetre, most of which are excitatory (85%) and the minority, inhibitory (15%). Unlike neurons in the retina, neurons in the visual cortex are preferentially activated by lines or edges of a particular orientation. This is termed a neuron's “orientation preference.” In the visual cortex of higher mammals like cats and monkeys, neurons that share an orientation preference are clustered in functional columns. However, in rodents like mice, orientation preferences are randomly distributed. In this study, we investigate whether the differences between columnar and non-columnar cortex is correlated with differences in the connectivity patterns between excitatory and inhibitory neurons. Using light and electron microscopy, we mapped the connectivity of pyramidal neurons—the primary excitatory neurons—in the superficial layers of the primary visual cortex (V1) of mice. Our results show that the ratio of excitatory-inhibitory neurons in mouse V1 is similar to that of cat or monkey V1, but in mouse V1 local pyramidal neurons target proportionately many more inhibitory neurons compared to what other studies found in cat or monkey. This difference may indicate the significance of inhibition in maintaining orientation selectivity in the non-columnar visual cortex of rodents like mice and is a distinct difference in the architecture of V1 between mice and higher mammals.

## Introduction

The concept of the cortical “column” is one of the few organising principles for cortical circuits that we have, yet the characteristic orientation columns in the primary visual cortex (V1) of the cat and monkey appear to be completely absent in rodent V1. In place of the ordered maps of orientation seen in cat and monkey, the distribution of orientation preferences in rodent V1 appears to be essentially random [1]–[3]. This “salt-and-pepper” arrangement in the rodent must reflect differences in the wiring of superficial layer neurons in rodents compared to cat and monkey.

Another striking difference between V1 of mouse and those of cat and monkey is the tuning properties of inhibitory neurons. While in cat and monkey the receptive fields of smooth (putative GABAergic inhibitory) neurons are typically orientation selective [4]–[10], with only occasional exceptions [11], in the mouse they are essentially weakly tuned [12],[13] (but see Runyan and colleagues [14]).

A third striking difference is that neurons in mouse V1 receive many more synapses on average [15],[16] than a neuron in primary visual cortex of cat [17],[18] or monkey [15],[19]–[21]. In rodent barrel cortex a significant proportion of these synapses are probably contributed by neighbouring pyramidal cells, which form their synapses on the basal dendrites [22],[23]. In cat, superficial layer pyramidal neurons are estimated to receive more than 60% of the excitatory synapses from their neighbouring pyramidal neurons [24]. This suggests that positive feedback loops are more likely between superficial layer pyramidal cells than between pyramidal cells in other layers, whose principal projections tend to project out of their home layers (see Douglas and Martin [25]). By implication, the large number of excitatory synapses per neuron in the mouse may require a stronger component of recurrent inhibition.

Clear evidence for an enhanced inhibitory component in the recurrent circuit came from a recent ultrastructural study by Bock et al. [26] designed to investigate whether the broadly tuned receptive fields of GABAergic inhibitory neurons could be explained by the convergence of input from excitatory neurons with different orientation preferences. This work involved partial reconstruction of 13 pyramidal cells and one smooth (putative GABAergic inhibitory) neuron in a single 50 µm thick section of V1 from a mouse that had undergone calcium imaging in vivo [26]. Their main conclusion was that pyramidal cells of different orientation preferences converged on individual smooth neurons. No synapses were formed between any of the 13 pyramidal cells.

A remarkable statistic from Bock et al. was that 51% of the synapses formed by the pyramidal axons were targeting smooth neurons. This is a staggeringly high proportion, and it implies a very different wiring strategy from the cat or monkey V1, where the proportion of excitatory synapses formed by layer 2/3 pyramidal neurons with smooth neurons is 5% [27] and 19% [28], respectively. The results of Bock et al. thus raise the question of whether this arises because there are proportionately more smooth neuron targets in the mouse, or whether pyramidal cells select smooth neurons as their targets in a way they do not in the cat or monkey.

To answer these questions we made detailed analyses not just of the synaptic targets of superficial layer pyramidal cells, but also of the content of the neuropil in mouse V1 in our material, and made the same analyses of the neuropil in the material of Bock et al. [26]. Our analyses indicate that the superficial pyramidal cells do not connect randomly to dendrites in the neuropil, as Braitenberg and Schüz [29] have proposed (and named “Peters' rule”), but instead form a far higher proportion of their synapses with neighbouring smooth neurons than would be expected by chance.

## Results

**Figure 1:**
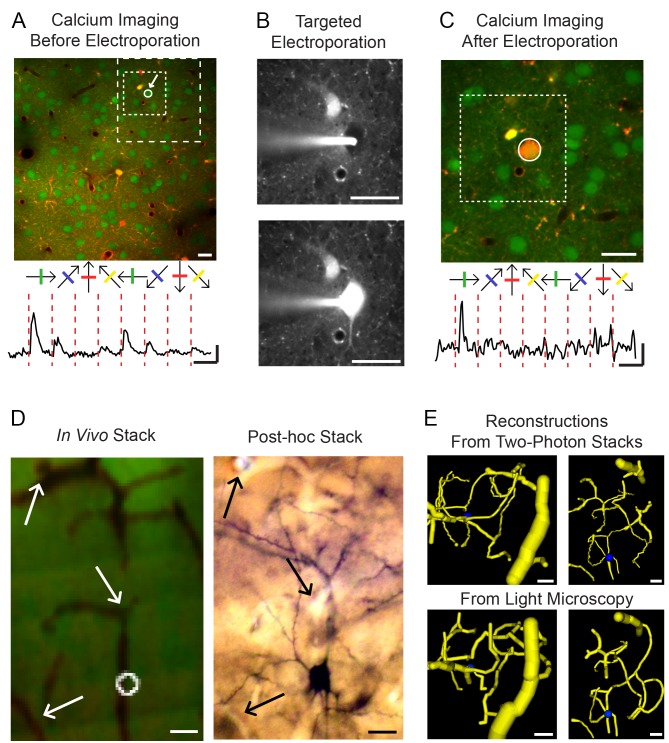
Targeted electroporation of functionally identified neurons in mouse V1. (**A**) Upper panel: Example two-photon image of V1 (286×286 µm field of view); neurons were labelled with the calcium indicator OGB-1/AM (green) and astrocytes with sulforhodamine (SR101, red). The dotted squares show the limits of the areas seen in (**B**) and (**C**). Lower panel: Averaged calcium signals of the selected neuron in response to drifting gratings. Scale bars: horizontal: 10 s, vertical: 20% ΔF/F. (**B**) Targeted electroporation of the neuron shown in (**A**). Upper panel: Two-photon image of the red channel before electroporation; the targeted neuron appears as a black hole. The glass pipette used for electroporation, filled with biocytin and Alexa Fluor 594 (see Materials and Methods) appears in white. Lower panel: Same picture after electroporation. (**C**) Calcium imaging after electroporation. The electroporated neuron contains OGB-1/AM and Alexa Fluor 594 and consequently appears in orange. Lower panel: Averaged calcium signals of the electroporated neuron. Scale bars: horizontal: 10 s, vertical: 5% ΔF/F. Notice that the neuron has kept its orientation tuning. The dotted square shows the limits of the area seen in (**B**). (**D**) Side view of blood vessels and the electroporated cell in a two-photon image stack *in vivo* (left) and in a post-hoc light microscopy stack (right). The arrows indicate recognizable common features between the two stacks. (**E**) Blood vessels reconstruction from the two-photon stack (upper panels) and the light microscopy stack (lower panels). Left pictures represent a top view of the reconstruction, right pictures are side views. Notice the similarity between the two-photon and the light microscopy reconstructions. All scale bars are 20 µm.

Our goal was to determine the proportion of pyramidal cell synapses with smooth and spiny neurons and to determine whether the composition of the neuropil reflected the proportion of pyramidal axon targets found experimentally. To replicate the results of Bock et al. [26], we used the same mouse strain and, as they did, used in vivo calcium imaging with two-photon microscopy (2PM) to record the responses of V1 neurons to visual stimulation. In contrast to the Bock study, however, we did not reconstruct the unlabelled axons by serial section electron microscopy (EM). Instead, we reduced the load of EM reconstruction considerably by filling individual imaged neurons with biocytin by electroporation after their functional characterization using 2PM, and making a correlated light/electron microscopic examination of their axons (Figure 1).

We successfully characterized the morphology and synaptic ultrastructure of six neurons in layer 2/3 of five mice. The visual tuning properties were obtained for five neurons in five mice. In one mouse, a second serendipitously filled neuron was also reconstructed. Figure 1 shows the steps from imaging to electroporation to recovering the functionally characterised neuron. After 2PM calcium imaging of the neuronal population and functional characterization using drifting gratings, a reliably responsive and selective neuron was selected for electroporation. The white arrow in Figure 1A shows the neuron selected, which was tuned for vertically oriented drifting gratings (Circular Variance Index [CVI] = 0.62; Direction Selectivity Index [DSI] = 0.17). We then used targeted electroporation to label this neuron (Figure 1B). We observed that the electroporated neuron maintained its selectivity and responsiveness (Figure 1C, Figure S1). By aligning a stack of 2PM images with the serial 80 µm thick sections imaged with bright field light microscopy (LM), the recorded neuron was identified and recovered for morphological examination (Figure 1D, E).

**Figure 2:**
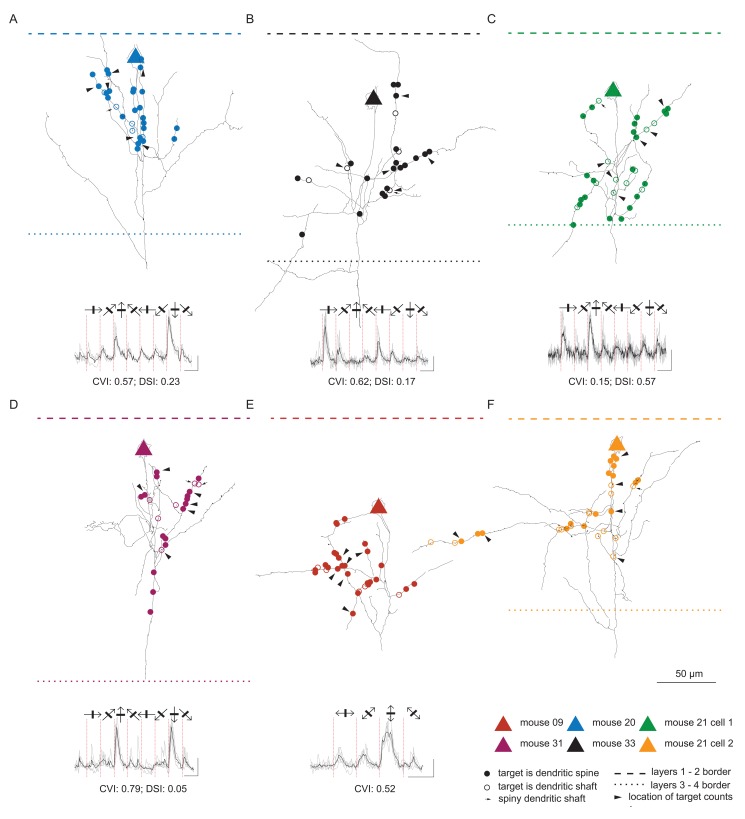
Structural and functional mapping of layer 2/3 pyramidal neurons. Each panel (**A–F**) shows the morphology and physiology of each of the single neurons analysed in this study. It contains a camera lucida drawing of the axonal arbour contained within a single 80 µm section (black lines). The triangles mark the location of the soma and the circles mark the location of the axonal varicosities investigated with light-electron correlated microscopy. The layer 1–2 border is displayed as a dashed line and the layer 3–4 border as a dotted line. The number of dendritic spine and shaft targets was counted in the neuropil surrounding the varicosities indicated with arrowheads. The response of the neurons to oriented gratings is displayed under the reconstruction together with the Circular Variance Index (CVI) and the Direction Selectivity Index (DSI). Black lines denote mean responses and gray lines individual trials.

Figure 2 shows the full extent of the axon contained within the single 80 µm thick section containing the cell body. The position of the soma and the distribution of the axons were very similar to those reconstructed by Bock et al. [26]. The position of the cell body relative to the laminae is indicated by a triangle and the laminar borders are indicated with dashed and dotted lines. The circles on the black axons show the positions of the boutons that formed synapses. Filled circles indicate synapses formed with spines and open circles indicate synapses formed with dendritic shafts. Arrowheads indicate boutons where the composition of the surrounding neuropil was analysed. The traces below each reconstruction are averages from calcium imaging and show that all five neurons were orientation tuned and/or directionally biased.

Serial ultrathin sections were taken through the axon to examine 21–31 boutons per neuron. These segments of the axon were correlated with the light microscopy (LM) reconstructions to define the precise position of the synapses and their targets along the axon. A total of 163 boutons were investigated in the EM. They formed a total of 170 synapses (148 boutons formed one synapse, 11 boutons formed two synapses, and four boutons formed no synapses).

**Figure 3:**
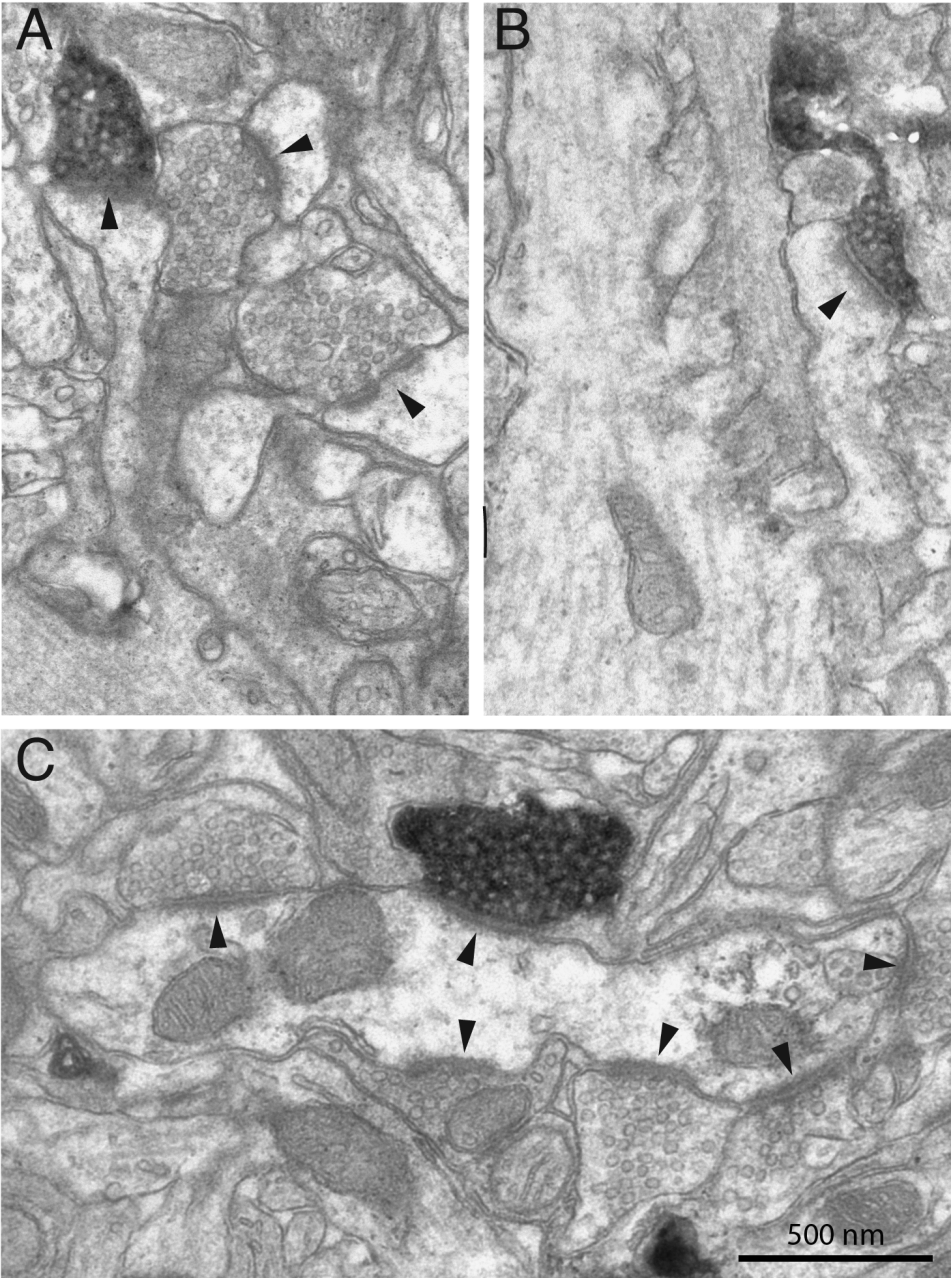
Electron micrographs of labelled boutons forming synapses with dendritic spines (A, B) and a dendritic shaft (C). Black arrowheads indicate synapses.

The different targets of the pyramidal axons were classified by standard criteria [27]. Figure 3 shows two examples of spines (Figure 3A, B) forming synapses with the biocytin-labelled boutons, which are electron-dense and filled with vesicles. The large postsynaptic density (arrow head) indicates a typical asymmetric synapse formed by pyramidal neurons. Two unlabelled vesicle-filled boutons forming asymmetric synapses are also indicated in Figure 3A (arrowheads) for comparison. In Figure 3C the bouton formed an asymmetric synapse with the dendritic shaft of a smooth neuron. Unlike the dendrites of spiny neurons, where most asymmetric synapses are formed with spines, the asymmetric synapses formed with dendrites of smooth (i.e. spine-free) neurons are naturally found on the shafts (arrow heads in Figure 3C). In all cases the classification of the targets was made on the basis of serial section analyses of the postsynaptic dendrite.

**Figure 4:**
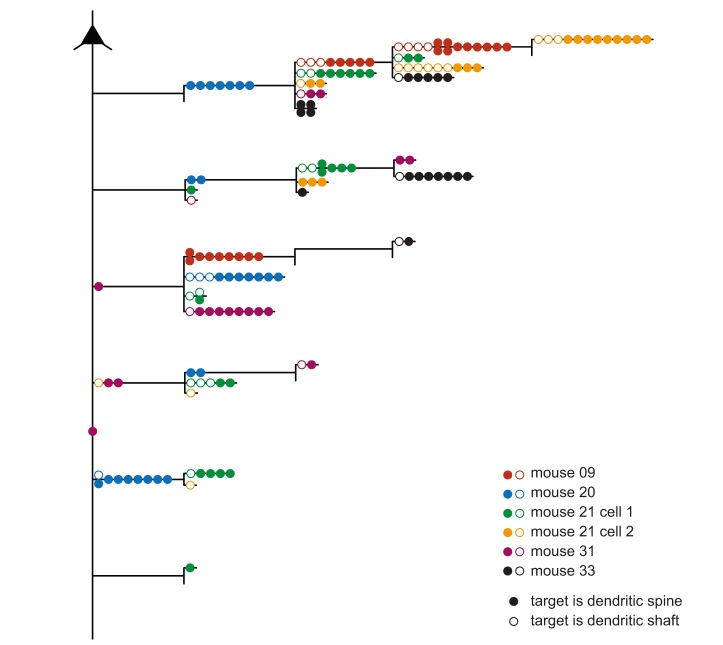
Summary dendrogram of layer 2/3 axon with the location of investigated synapses per branch order. Circles indicate the location of the investigated synaptic boutons. Colours represent different neurons; targeting of dendritic spines is indicated by filled circles and dendritic shafts by empty circles.

By reconstructing the axons at the LM level, we were able to identify the particular branch segments that contained the synapses examined with subsequent EM. Figure 4 shows a summary dendrogram that reveals the branch ordering of the axons and the relative location of the 170 synapses examined on the axons. As in Figure 2, the target type is indicated by closed circles for spines and open circles for dendritic shafts. The axon leaving the soma descends vertically before branching and forming collaterals with boutons in layers 2 and 3. The dendrogram shows that the synapses we sampled were found on all orders of the branches, even on the main descending axon.

**Figure 5:**
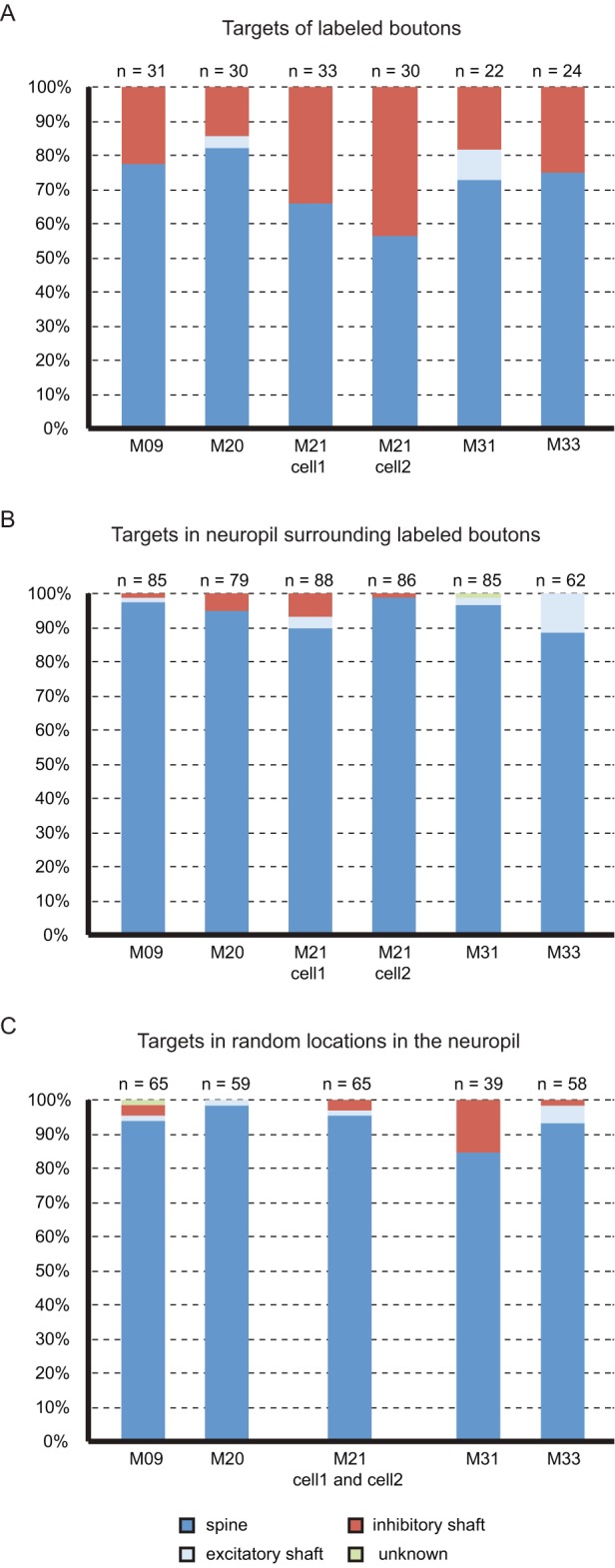
Histograms showing the percentage of the different types of post-synaptic targets of (A) layer 2/3 labelled neurons, (B) unlabelled boutons in the neuropil surrounding labelled boutons, and (C) unlabelled boutons from random locations in the neuropil. Targets were considered as smooth dendritic shaft, spiny dendritic shaft, and dendritic spine.

A total of 126 synapses were formed with spiny neurons (120 formed with dendritic spines and 6 with dendritic shafts) and 44 with dendritic shafts of smooth neurons. The data for each neuron in terms of target type are plotted in the histograms of Figure 5A. These histograms show that although spines formed the majority of targets, the variance between individual neurons was surprisingly high. If Peters' rule [29] applied, we would expect the proportion of different targets to reflect the local average proportions of smooth and spiny neurons in layer 2/3. The question was whether this high variance reflected some local heterogeneities in distribution of targets in the neuropil, or whether it was due to specific targeting of smooth neurons by some pyramidal cells. We tested this using the unbiased disector counting technique (see Materials and Methods [30],[31]) to determine the distribution of asymmetric synapses formed with spiny or smooth neurons in the neuropil at the vicinity of each of the reconstructed neurons. These results show that in the neuropil many more synapses were formed with spines than were found for the labelled axons (Figure 5C). Next, we explored the possibility that the observed specificity was due to the fact that labelled boutons formed synapses in regions of the neuropil where there were more dendritic shaft targets. Again using the physical disector method we placed a 5 µm×5 µm sampling square centred on labelled boutons (randomly selected boutons indicated by arrowheads in Figure 2). The results plotted in Figure 5B show that in the region around any labelled bouton, virtually all the synapses were formed with spines. This was again different from the distribution of targets of the labelled boutons, which formed significantly more synapses with smooth neurons than Peters' rule would predict.

Finally, to test whether the difference in targeting between labelled axons and the unlabelled neuropil could be due to a random process, we ran a simulation of an axon growing through a virtual neuropil and connecting to its targets by chance. The location of targets in the neuropil was uniformly distributed, as found in the large disectors (30 µm×30 µm), and each simulation was performed 10,000 times. When the simulations used the percentage of smooth dendritic targets collected from locations surrounding labelled boutons (Figure 5B), the Monte Carlo analysis (Figure S2) revealed that, with the exception of neuron M20 (*p* = 0.077) the other neurons showed a strong statistical difference (*p* = 0) between the number of inhibitory targets observed experimentally and that predicted from a random process. When the simulations used the percentage of smooth dendritic targets collected from random locations in the neuropil (Figure 5C), the Monte Carlo analysis (Figure S3) revealed that, with the exception of neuron M31 (*p* = 0.48), the other neurons showed strong a statistical difference (*p* = 0) between the number of inhibitory targets observed experimentally and that predicted from a random process.

**Figure 6:**
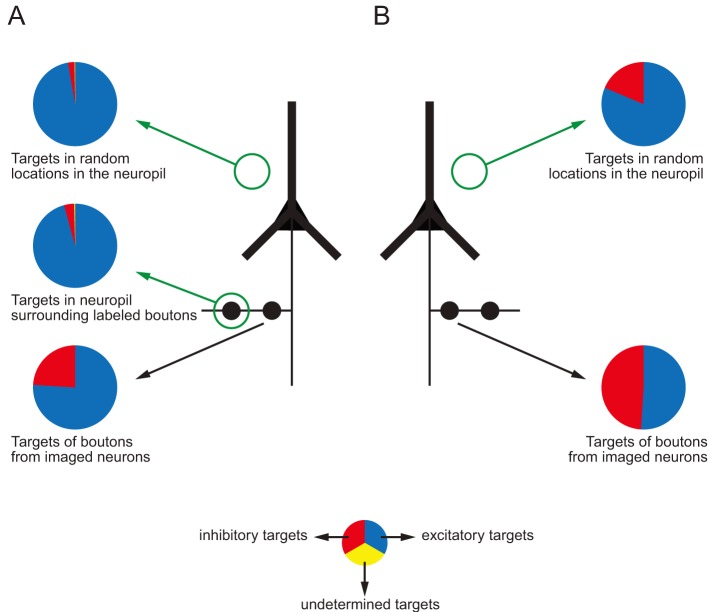
(A) Summary diagram of the distribution of post-synaptic targets in layer 2/3 of mouse visual cortex. (B) Comparison with the study of Bock et al. (2011) [26].

We also tested whether the biases observed by Bock et al. [26] followed the same trend as our data. We applied the unbiased disector method on their web-based data to estimate the proportion of synapses formed with spiny and smooth neurons in the neuropil of the superficial cortical layers of their mouse (pie charts in Figure 6, right column). We found that 80% of the targets were on spiny dendrites (35 synapses on spines and one on a spiny shaft) and 20% on smooth dendrites (nine synapses). Our analyses of their data indicate that the axons contained in their reconstructed volume targeted far more smooth neurons than would be expected from the composition of the neuropil through which they passed (compare Figure 6, data from imaged neurons in the lower pie chart with disector counts in the upper pie chart). Thus, although on average our labelled neurons formed proportionately fewer synapses with smooth neurons than did those of Bock et al. [26], in both studies the proportion of targeted smooth neurons was far higher than would be expected on the basis of random connectivity. Thus the data from both studies indicate that these superficial layer pyramidal cells in mouse V1 appear to select smooth neurons as their targets.

## Discussion

Our goal was to establish whether the salt-and-pepper representation of orientation in rodent V1 is reflected in the synaptic connections formed by the superficial layer pyramidal cells. After 2PM calcium imaging, individual pyramidal neurons were labelled with biocytin, sectioned, and reconstructed with LM. The synaptic targets of their axons as well the synaptic complement of the surrounding neuropil were quantified using EM. Previous physiological studies suggested that pyramidal cells connect specifically to one another [32]–[36] and to GABAergic neurons [37]. Moreover, in mouse V1, the probability of pyramidal neurons connecting to neighbouring fast-spiking interneurons is much higher than the probability of pyramidal neurons connecting to each other [38]. Our data further indicate that pyramidal cells make specific connections with smooth, putative GABAergic neurons.

A previous combined 2PM calcium imaging and electron microscopy study by Bock et al. [26] of 13 pyramidal cells in one mouse indicated that the pyramidal cells formed a consistently high proportion (50%) of their synapses with smooth, putative GABAergic neurons. This is an astonishingly high fraction, since more extensive analyses of superficial layer pyramidal cells in V1 of other species indicate that typically 20% or fewer of the synapses are formed on smooth neurons. One explanation for the data from Bock et al. might be that the neuropil of mouse V1 contains a higher proportion of smooth neurons than other species. This seems not to be the explanation since no major differences have been noted in the proportion of pyramidal cells and smooth neurons in the superficial layers of rodent, cat, or monkey V1 [20],[39],[40]. The critical question is then whether the result obtained from the single section in one mouse by Bock et al [26] is an outlier, or whether it really reflects a wiring strategy to increase the local component of recurrent inhibition in V1.

The composition of the neuropil based on our own samples and those of Bock et al. [26] indicates that the superficial layer pyramidal cells in mouse V1 form a significantly higher proportion of their synapses with smooth, putative inhibitory neurons than would be predicted by Peters' rule [29], which assumes that axons and dendrites connect in the proportions in which they are found in the neuropil. Our disector counts indicated that virtually all the unlabelled synapses in the neuropil within a 5 µm radius of any of the labelled pyramidal cell boutons were formed with spines, not smooth dendrites. Bock et al. [26] concluded that geometry dominates over function, since the proximity of two pyramidal cells, not their receptive field similarity, was the strongest indicator that their axons would converge onto a smooth neuron. However, our own results, and our new analyses of the cortical tissue from Bock et al., indicates that the pyramidal cell connections to smooth neurons are far from being determined purely by geometry, for if geometry were the sole determinant, the pyramidal neurons should connect to smooth neurons in proportion to their occurrence in the neuropil. Instead, some pyramidal cells preferentially formed a subset of their synapses with smooth neurons. What is unexplained, however, is why the variance across the pyramidal cells is so high. In this context it is noteworthy that all the pyramidal cells in the mouse of Bock et al. had consistently high proportions of smooth targets, as did the mouse in which we examined two pyramidal cells. This suggests that the source of variance might not be within the individual, but between individuals of the same strain. These interesting observations across the two studies raise both a warning and an interesting challenge as to how we might discover the principles by which mouse brain wires itself if such high variance does exist between individuals.

**Figure 7:**
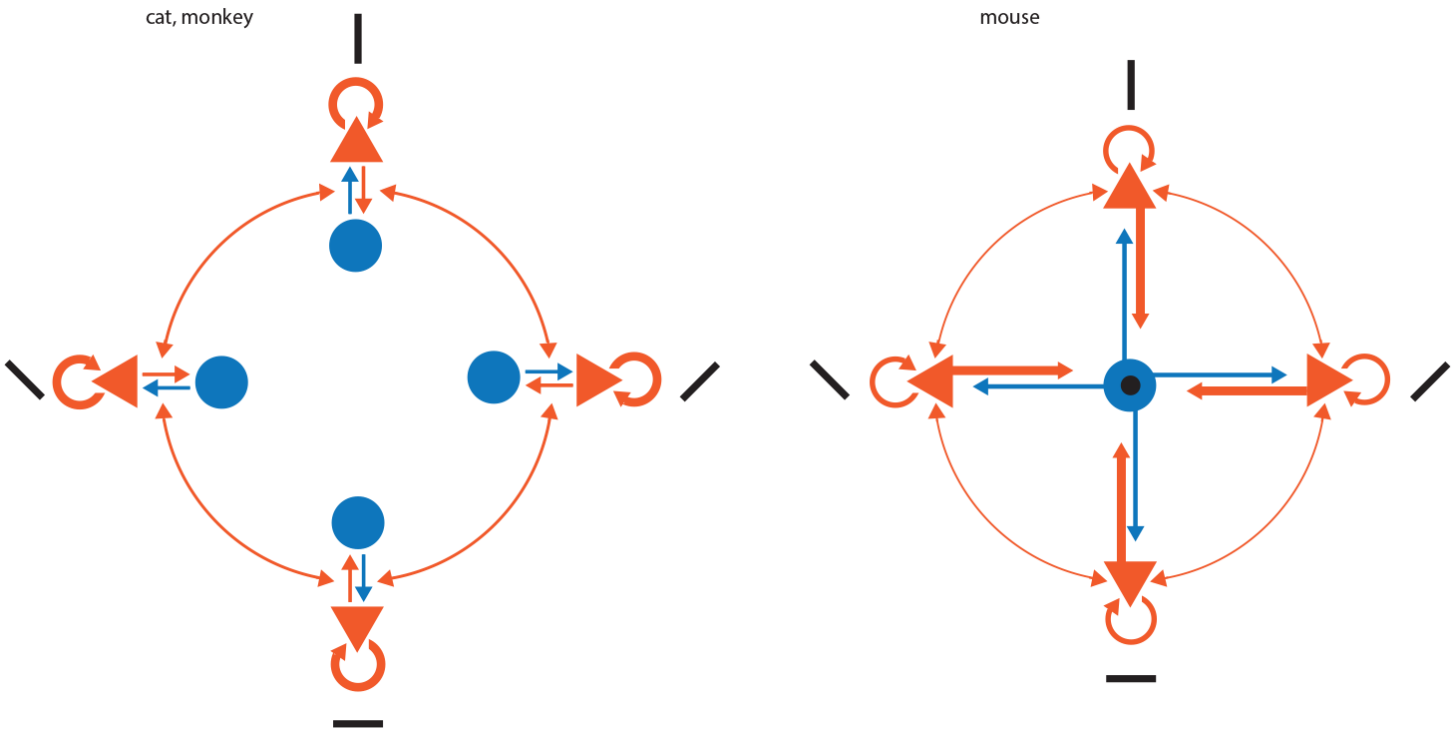
Comparison between the circuit of layer 2/3 of visual cortex of cat and monkey with the circuit of the mouse. Inhibitory neurons are represented as blue disks and excitatory neurons as orange triangles. Arrows indicate synaptic connections, and the thickness of the arrow represents its weight in terms of number of connections. Black bars represent the orientation preference of each of the neurons, and a black disk indicates an untuned or poorly tuned neuron.

Our results and those of Bock et al. have implications for the functional architecture of mouse visual cortex (Figure 7) and its operation. If layer 2/3 smooth neurons receive more than their fair share of synapses from local pyramidal cells than would be expected from Peters' rule, this implies that they receive proportionally fewer synapses from other excitatory projections into layers 2 and 3. These other excitatory inputs arise from spiny neurons in layer 4 and 5 of V1 as well as other cortical areas and subcortical nuclei, like the thalamus. By this argument, pyramidal cells then have proportionately fewer synapses to devote to connections to other pyramidal neurons in the same layer. If this is the case, then in layer 2/3 of mouse visual cortex one might expect proportionately less recurrent excitation from within these layers than is present in the cat.

Smooth neurons, like basket cells and chandelier cells, form their axonal arbours largely within the same layer as the cell body. Therefore, the smooth neurons targeted by our labelled pyramidal cell axons most likely are recurrently inhibiting the pyramidal cells that excite them. The fact that in mouse the smooth neurons have more convergent input from neurons with a variety of orientation tuning produces a circuit configuration that is very reminiscent of a winner-take-all (WTA) circuit. In this circuit, excitatory neurons have a map of some parameter (e.g. orientation [35],[36]), and the inhibitory neurons receive input from all excitatory neurons in the map and provide inhibition proportional to the overall excitation in the circuit [41].

While this study focuses on mouse V1, previous work on superficial layer pyramidal cells in V1 of cat and monkey gave dramatically different results to those presented here. In monkey V1, McGuire et al. [28] have shown that the axons of intracellularly filled layer 2/3 pyramidal neurons formed 19% of their targets with smooth dendritic shafts (28% if one considers spiny and dendritic shafts). As we did, McGuire et al. [28] analysed the neuropil surrounding one of their neurons, but unlike us found no evidence for preferential targeting of smooth GABAergic neurons by the superficial layer pyramidal neurons (see also Beaulieu and colleagues for other counts of targets in monkey V1 neuropil [20]).

In cat V1, Kisvarday et al. [27] found that the axons of intracellularly filled layer 3 pyramidal neurons formed only 5% of their synapses with GABAergic neurons. They did not analyse the neuropil surrounding the labelled neurons, but in a different study Beaulieu and Colonnier analysed the neuropil of cat layer 2/3 of and found that 18% (mean of layers 2, 3A, and 3B) of the asymmetric synapses are formed with dendritic shafts, some of which may be of spiny neurons [18]. These data strongly suggested that in cat there is no preferential targeting of inhibition by layer 2/3 pyramidal neurons, unlike what we, and Bock et al. [26], now find for mouse V1. This was also the conclusion of a theoretical study by Stepanyants and colleagues [42], who found that the results of Kisvarday et al. [27] were consistent with Peters' rule. The conclusion of Stepanyants et al. makes it very clear that in the cat the proportion of GABAergic smooth neurons that are targets of superficial layer pyramidal axons is well below that of the mouse V1.

One idea for the generation of orientation “columns” in cat is that the orientation selectivity of neurons is created in layer 4 and then simply fed-forward to neurons in the superficial and deep layers [43]. In the macaque monkey, the situation is somewhat different, because most layer 4C neurons have non-oriented receptive fields, whereas neurons in the superficial and deep layers are orientation selective and form an orderly map of orientation, as in the cat. Development of the acolumnar salt-and-pepper arrangement of rodent V1 demands a high degree of specificity if it were to be achieved by feed-forward connections alone. Here, the stronger bias in the connections to smooth cells in the mouse may reflect increased demands on the inhibitory circuitry to shape the receptive field mediated by the superficial layer pyramidal cells.

## Materials and Methods

### Animal Preparation

All animal procedures were carried out according to the guidelines of the University of Zurich, and were approved by the Cantonal Veterinary Office. C57BL/6 mice (2–4 months old, of either sex) were either first sedated with chlorprothixene (Sigma; 0.2 mg/mouse) and anaesthetized with urethane (0.5–1.0 g/kg) or anaesthetized by 2.7 ml/kg of a solution containing one part fentanyl citrate and fluanisone (Hypnorm; Janssen-Cilag, UK) and one part midazolam (Hypnovel; Roche, Switzerland) in two parts of water, both delivered by intraperitoneal injections. Atropine (0.3 mg/kg) and dexamethasone (2 mg/kg) were administered subcutaneously to reduce secretions and oedema. Lactate-Ringer solution was regularly injected subcutaneously to prevent dehydration. Pinch reflexes were used to assess the depth of anaesthesia.

### Two-Photon Guided Staining

The location of the primary visual area, V1, was determined by stereotaxic coordinates (V1 monocular segment – 1.0 mm anterior to lambda and 2.5 mm lateral from the midline [44]) and confirmed by subsequent intrinsic imaging. Briefly, the skull above the estimated visual cortex was carefully thinned until a noticeable transparency of the bone was achieved. We then illuminated the cortical surface with 630-nm LED light, presented drifting gratings for 5 s, and collected reflectance images through a 4× objective with a CCD camera (Toshiba TELI CS3960DCL). Intrinsic signal changes were analysed as fractional reflectance changes relative to the pre-stimulus average. V1 was the largest area active during visual stimulation at a location in accordance with stereotaxic coordinates.

After identification with intrinsic imaging, a small craniotomy (from 500 µm×500 µm to 1 mm×1 mm) was opened above V1, the dura removed and the exposed cortex superfused with artificial cerebrospinal fluid (ACSF) (135 mM NaCl, 5.4 mM KCl, 5 mM Hepes, 1.8 mM CaCl_2_, 1 mM MgCl_2_, pH 7.2, with NaOH). Calcium indicator loading was performed using the “multi cell bolus loading” technique [45]. Briefly, 50 µg of the acetoxymethyl (AM) ester form of the calcium-sensitive fluorescent dye Oregon Green BAPTA-1 (OGB-1; Invitrogen, Basel, Switzerland) were dissolved in 2 µl of DMSO plus 20% Pluronic F-127 (BASF, Germany) and diluted with 37 µl standard pipette solution (150 mM NaCl, 2.5 mM KCl, 10 mM Hepes, pH 7.2) yielding a final OGB-1 concentration of about 1 mM. 1 µl of Alexa Fluor 594 (Invitrogen; 2 mM stock solution in distilled water) was added for visualization of the pipette during 2PM guided staining. The dye was pressure ejected under visual control through a glass pipette (4–5 MΩ) at a depth between 150–300 µm to stain layer 2/3 neurons. Brief application of sulforhodamine 101 (SR101; Invitrogen) to the exposed neocortical surface resulted in co-labelling of the astrocytic network [46]. Following dye injection the craniotomy was filled with agarose (type III-A, Sigma; 1% in ACSF) and covered with an immobilized glass cover slip.

### Visual Stimulation

Visual stimuli were presented on a 7-inch TFT monitor (75 Hz refresh rate) 7 cm in front of the right eye roughly at 60° along the body axis of the anesthetized mouse. For the majority of the study, the visual stimuli were full contrast square wave gratings generated by the VisionEgg software [47] moving for 3 s in eight different directions spaced by 5 s blank (grey screen presentation). The temporal frequency (TF) was 0.5 to 1 Hertz (Hz) and spatial frequency (SF) was 0.02 to 0.05 cycles per degree (cyc/°), which have been shown to activate most neurons. For one animal, the stimulation used was full contrast square wave gratings moving back and forth during 4 s for each of four orientations.

### Two-Photon Calcium Imaging

Calcium transients were acquired using a custom-built two-photon microscope equipped with a 40× water immersion objective (LUMPlanFl/IR; 0.8 NA; Olympus 2). 128×128 pixel frames or 256×256 pixel frames were acquired at rates from 2 to 4 Hz using custom written software (LabView; National Instruments, USA).

### Calcium Signal Analysis

Data were analysed with ImageJ (National Institute of Mental Health, NIH) and MATLAB (Mathworks). Cells were detected manually by drawing a region of interest around cell bodies. Relative percentage changes in fluorescence (ΔF/F) were calculated using as baseline the blank just before each stimulation. Traces were filtered using a Savitzky-Golay filtering approach. Responses were calculated by averaging 3–6 points around the peak fluorescence change (time window of 1.5 s around the peak) for each stimulation epoch.

### Circular Variance

We defined a selectivity criterion using circular variance over gratings responses. Circular variance is defined as 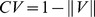, where *θ* is the average drift direction of the grating:
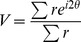



This measure of circular variance combines aspects of amplitude modulation and tuning width and takes into account all the responses to each direction of drift [48]. To use it as an index comparable to orientation selectivity indexes, the values given in this manuscript are 1 – circular variance (see Niell and Stryker [49]) referred to in the text as CVI, for Circular Variance Index). Consequently, a perfectly tuned neuron would have a CVI value close to 1, and a perfectly untuned neuron close to 0. For further analysis of selectivity we used the Direction Selectivity Index (DSI; see below).

### Direction Selectivity

We determined the direction selectivity as previously described [48],[49]. It is defined as:
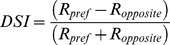
Where *R_pref_* is the response at the preferred angle *θ_pref_* and *R_opposite_* is the responses at the opposite direction *θ_pref_*+*π*. If DSI >0.5, the neuron is considered direction selective.

### Targeted Electroporation

Glass pipettes of resistance from 4 to 6 MΩ were filled with a standard pipette solution containing 2%–5% biocytin. These concentrations of biocytin were reached by mixing 4% biocytin (ε-Biotinoyl-L-Lysine; Invitrogen) diluted in some cases with the red dye Alexa 594 (20 µM; Invitrogen) with a solution of 0.8 to 1.5% 5-(and-6)-Tetramethylrhodamine biocytin (Biocytin TMR; Invitrogen).

The tip of the pipette was placed near the selected neuron for electroporation and a loose seal was formed to record extracellular spikes. Spikes were recorded at 5 kHz using a patch-clamp amplifier (npi, Reutlingen, Germany) and Spike2 software (CED, Cambridge, UK). Once a stable configuration was reached, pulses from 300 to 400 mV of 10 ms duration were applied until successful electroporation was verified visually by uptake of the red indicator dye. In addition, we verified in some experiments the viability of the neuron by retesting the responses to visual stimulation after a recovery period of 10–20 min. This recovery period allows sufficient time for the pores formed during the electroporation to reseal, which usually occurs within 1 min [50].

### Perfusion and Histology

At the end of the experiment the mouse was given an extra dose of anaesthesia and perfused transcardially with normal 0.9% NaCl solution, followed by a warm solution of 4% paraformaldehyde (w/v), 0.5% glutaraldehyde (v/v) and 15% saturated solution of picric acid (v/v) in 0.1 M PB pH 7.4. After fixation the mouse was perfused with solutions of 10%, 20%, and 30% sucrose in 0.1 M phosphate buffer (PB). Once the brain was removed it was allowed to sink in a 30% sucrose solution in 0.1 M PB to provide cryoprotection and then freeze-thawed in liquid nitrogen. The brains were then washed in 0.1 M PB for at least 2 h to allow them to recover from the shrinkage provoked by the incubation in sucrose solution. Sections containing V1 were cut at 80 µm in the coronal plane and collected in 0.1 M PB. After cutting, the sections were washed several times in buffer in order to remove any remaining fixative. To reveal biocytin the sections were washed in TBS and then incubated overnight (5°C) with an avidin-biotin complex (Vector ABC kit – Elite). The peroxidase activity was identified using 3-diaminobenzidine tetrahydrochloride (DAB) with nickel intensification. After assessment by LM, regions of tissue containing the imaged area were treated with 1% osmium tetroxide in 0.1 M PB, dehydrated through alcohols (1% uranyl acetate in the 70% alcohol) and propylene oxide, and flat mounted in Durcupan (Fluka) on glass slides.

### Postmortem Light and Electron Correlated Microscopy

Serial light micrographs were taken from the osmicated sections at different magnifications, and the blood vessel pattern surrounding labelled neurons was reconstructed using TrakEM2 [51]. A similar blood vessel reconstruction was done on the 2PM stacks acquired in vivo. These reconstructions were used to find the recorded neurons in the osmicated histological sections. Finally the micrographs taken from histological sections were superimposed on the 2PM images to confirm the correspondence of the recorded neurons.

The dendritic arbour and the proximal axon the neurons of interest were then reconstructed first in 2D using a drawing tube attached to a light microscope, and then in 3D from serial light micrographs using TrakEM2 [51].

Afterward, the tissue was serially resectioned at 50 nm thickness and collected on Pioloform-coated single slot copper grids. The axons of labelled neurons were then found in the ultrathin sections, and synapse connectivity between labelled axons and neuropil targets investigated with transmission electron microscopy (TEM). Synapses and associated structures were classified using conventional criteria [52],[53].

### Counts of Dendritic Targets

Estimations of the percentage of dendritic targets (spines or shafts) were performed at the EM level using the physical disector method [30]. The disector was composed of two serial sections of known thickness (50 nm) separated by one intervening section. Synapses that disappeared from reference to lookup section were counted and the target was classified as dendritic spine or shaft as in [54]. Both sections were used as reference and lookup doubling the number of disectors per site. Electron micrographs were collected at a magnification of (13,500×, pixel size 2.5 nm) with a digital camera (11 megapixels, Morada, Soft Imaging Systems). Four sets of counts were performed. The first set was done on randomly selected location in the neuropil surroundings the labelled neurons. The disectors had a size of 5 µm×5 µm and were sampled from the first intact section of every fourth grid (each grid contained eight sections on average). The sampling sites (five sites per animal) and grids were selected according to a systematic random sampling scheme [31],[55]. The second set was done on the neuropil surrounding the labelled boutons of recorded neurons. The counts were done in six randomly selected boutons per neuron and the disectors had a size of 5 µm×5 µm. The third set was from a single animal and the exact 2D location of the synapses was also collected for use in the Monte Carlo simulations described below. Three randomly located large disectors (size 30 µm×30 µm) were collected. The fourth set was collected from the dataset of Bock et al. [26] which was made available through CATMAID [56]. The disectors had a size of 12.7 µm×6.8 µm and the sampling sites (eight sampling sites) and sections were chosen according to a systematic random sampling scheme.

### Monte Carlo Simulations

A Monte Carlo analysis was performed to test whether the observed statistics of synaptic targets by labelled axons could be due to a random process. We ran a simulation in MATLAB (Mathworks) of an axon growing through a virtual neuropil of size 200 µm×200 µm×200 µm. Each simulation was run 10,000 times with the parameters from each labelled neuron/neuropil and was terminated when the virtual axon reached the number of synapses reconstructed for each labelled neuron. The result of each simulation was the proportion of smooth dendritic targets on the virtual axon.

The location of targets in the virtual neuropil followed a uniform distribution as found in the biological data obtained from three large disectors (30 µm×30 µm). The proportion of spiny and smooth dendritic targets in the virtual neuropil were taken from the counts shown in Figure 5 and the density of synapses used was 10^9^ synapses/mm^3^ following the work of Schüz and Palm [16]. The *p-*value estimate was given by the proportion of simulations that showed results larger than or equal to the measurements made in the real neurons.

## Supporting Information

Figure S1 Examples of responses before and after electroporation for the cells M20 and M21. Black traces are the averaged responses to drifting gratings. Stimulation onsets are indicated by orange dotted lines.
(TIF)

Figure S2 Histograms showing the distribution of the percentage of targets formed by virtual layer 2/3 axons with smooth dendrites obtained with Monte Carlo simulations. The percentage of the available smooth dendritic targets in the neuropil was taken from the disectors collected from locations surrounding labelled boutons (Figure 5B).
(EPS)

Figure S3 Histograms showing the distribution of the percentage of targets formed by virtual layer 2/3 axons with smooth dendrites obtained with Monte Carlo simulations. The percentage of the available smooth dendritic targets in the neuropil was taken from the disectors collected from random location in the neuropil (Figure 5C).
(EPS)

## Abbreviations

2PM: two-photon microscopy
CVI: Circular Variance Index
DSI: Direction Selectivity Index
EM: electron microscopy
LM: light microscopy

## References

[1] Girman S, Sauve Y, Lund R (1999) Receptive field properties of single neurons in rat primary visual cortex. J Neurophysiol 82: 301–311. 10400959 10.1152/jn.1999.82.1.301

[2] Ohki K, Chung S, Ch'ng Y, Kara P, Reid R (2005) Functional imaging with cellular resolution reveals precise micro-architecture in visual cortex. Nature 433: 597–603. 15660108 10.1038/nature03274

[3] Roth MM, Helmchen F, Kampa BM (2012) Distinct functional properties of primary and posteromedial visual area of mouse neocortex. J Neurosci 32: 9716–9726. 22787057 10.1523/JNEUROSCI.0110-12.2012 PMC6622284

[4] Gilbert CD, Wiesel TN (1979) Morphology and intracortical projections of functionally characterised neurones in the cat visual cortex. Nature 280: 120–125. 552600 10.1038/280120a0

[5] Martin K, Somogyi P, Whitteridge D (1983) Physiological and morphological properties of identified basket cells in the cat's visual cortex. Exp Brain Res 50: 193–200. 6641854 10.1007/BF00239183

[6] Somogyi P, Kisvárday ZF, Martin KA, Whitteridge D (1983) Synaptic connections of morphologically identified and physiologically characterized large basket cells in the striate cortex of cat. Neuroscience 10: 261–294. 6633861 10.1016/0306-4522(83)90133-1

[7] Kisvárday ZF, Martin KA, Whitteridge D, Somogyi P (1985) Synaptic connections of intracellularly filled clutch cells: a type of small basket cell in the visual cortex of the cat. J Comp Neurol 241: 111–137. 4067011 10.1002/cne.902410202

[8] Cardin JA, Palmer LA, Contreras D (2007) Stimulus feature selectivity in excitatory and inhibitory neurons in primary visual cortex. J Neurosci 27: 10333–10344. 17898205 10.1523/JNEUROSCI.1692-07.2007 PMC3025280

[9] Ahmed B, Anderson J, Martin K, Nelson J (1997) Map of the synapses onto layer 4 basket cells of the primary visual cortex of the cat. J Comp Neurol 380: 230–242. 9100134

[10] Anderson JC, Martin KA, Whitteridge D (1993) Form, function, and intracortical projections of neurons in the striate cortex of the monkey Macacus nemestrinus. Cereb Cortex 3: 412–420. 8260809 10.1093/cercor/3.5.412

[11] Hirsch JA, Martinez LM, Pillai C, Alonso J-M, Wang Q, et al (2003) Functionally distinct inhibitory neurons at the first stage of visual cortical processing. Nat Neurosci 6: 1300–1308. 14625553 10.1038/nn1152

[12] Kerlin AM, Andermann ML, Berezovskii VK, Reid RC (2010) Broadly tuned response properties of diverse inhibitory neuron subtypes in mouse visual cortex. Neuron 67: 858–871. 20826316 10.1016/j.neuron.2010.08.002 PMC3327881

[13] Liu B-H, Li P, Li Y-T, Sun YJ, Yanagawa Y, et al (2009) Visual receptive field structure of cortical inhibitory neurons revealed by two-photon imaging guided recording. J Neurosci 29: 10520–10532. 19710305 10.1523/JNEUROSCI.1915-09.2009 PMC2779138

[14] Runyan CA, Schummers J, Van Wart A, Kuhlman SJ, Wilson NR, et al (2010) Response features of parvalbumin-expressing interneurons suggest precise roles for subtypes of inhibition in visual cortex. Neuron 67: 847–857. 20826315 10.1016/j.neuron.2010.08.006 PMC2948796

[15] Cragg B (1967) The density of synapses and neurones in the motor and visual areas of the cerebral cortex. J Anat 101: 639–654. 4964696 PMC1270900

[16] Schüz A, Palm G (1989) Density of neurons and synapses in the cerebral cortex of the mouse. J Comp Neurol 286: 442–455. 2778101 10.1002/cne.902860404

[17] Beaulieu C, Colonnier M (1983) The number of neurons in the different laminae of the binocular and monocular regions of area 17 in the cat, Canada. J Comp Neurol 217: 337–344. 6411777 10.1002/cne.902170308

[18] Beaulieu C, Colonnier M (1985) A laminar analysis of the number of round-asymmetrical and flat-symmetrical synapses on spines, dendritic trunks, and cell bodies in area 17 of the cat. J Comp Neurol 231: 180–189. 3968234 10.1002/cne.902310206

[19] O'Kusky J, Colonnier M (1982) A laminar analysis of the number of neurons, glia, and synapses in the adult cortex (area 17) of adult macaque monkeys. J Comp Neurol 210: 278–290. 7142443 10.1002/cne.902100307

[20] Beaulieu C, Kisvarday Z, Somogyi P, Cynader M, Cowey A (1992) Quantitative Distribution of GABA-immunopositive and-immunonegative Neurons and Synapses in the Monkey Striate Cortex (Area 17). Cerebral Cortex 2: 295–309. 1330121 10.1093/cercor/2.4.295

[21] Mouton PR, Price DL, Walker LC (1997) Empirical assessment of synapse numbers in primate neocortex. Journal of neuroscience methods 75: 119–126. 9288643 10.1016/s0165-0270(97)00058-7

[22] Koester HJ, Johnston D (2005) Target cell-dependent normalization of transmitter release at neocortical synapses. Science 308: 863–866. 15774725 10.1126/science.1100815

[23] Feldmeyer D, Lubke J, Sakmann B (2006) Efficacy and connectivity of intracolumnar pairs of layer 2/3 pyramidal cells in the barrel cortex of juvenile rats. J Physiol (Lond) 575: 583–602. 16793907 10.1113/jphysiol.2006.105106 PMC1819447

[24] Binzegger T, Douglas R, Martin K (2004) A quantitative map of the circuit of cat primary visual cortex. J Neurosci 24: 8441–8453. 15456817 10.1523/JNEUROSCI.1400-04.2004 PMC6729898

[25] Douglas R, Martin K (2004) Neuronal circuits of the neocortex. Annu Rev Neurosci 27: 419–451. 15217339 10.1146/annurev.neuro.27.070203.144152

[26] Bock DD, Lee W-CA, Kerlin AM, Andermann ML, Hood G, et al (2011) Network anatomy and in vivo physiology of visual cortical neurons. Nature 471: 177–182. 21390124 10.1038/nature09802 PMC3095821

[27] Kisvárday ZF, Martin KA, Freund TF, Maglóczky Z, Whitteridge D, et al (1986) Synaptic targets of HRP-filled layer III pyramidal cells in the cat striate cortex. Exp Brain Res 64: 541–552. 3803491 10.1007/BF00340492

[28] McGuire BA, Gilbert CD, Rivlin PK, Wiesel TN (1991) Targets of horizontal connections in macaque primary visual cortex. J Comp Neurol 305: 370–392. 1709953 10.1002/cne.903050303

[29] Braitenberg V, Schüz A (1991) Anatomy of the cortex. 2nd ed. Berlin: Springer-Verlag.

[30] Sterio D (1984) The unbiased estimation of number and sizes of arbitrary particles using the disector. J Microsc 134 Pt 2: 127–136. 6737468 10.1111/j.1365-2818.1984.tb02501.x

[31] Da Costa NM, Hepp K, Martin KAC (2009) A systematic random sampling scheme optimized to detect the proportion of rare synapses in the neuropil. J Neurosci Methods 180: 77–81. 19427532 10.1016/j.jneumeth.2009.03.001

[32] Yoshimura Y, Dantzker JLM, Callaway EM (2005) Excitatory cortical neurons form fine-scale functional networks. Nature 433: 868–873. 15729343 10.1038/nature03252

[33] Kampa BM, Letzkus JJ, Stuart GJ (2006) Cortical feed-forward networks for binding different streams of sensory information. Nat Neurosci 9: 1472–1473. 17099707 10.1038/nn1798

[34] Song S, Sjöström PJ, Reigl M, Nelson S, Chklovskii DB (2005) Highly nonrandom features of synaptic connectivity in local cortical circuits. PLoS Biol 3: e68. 15737062 10.1371/journal.pbio.0030068 PMC1054880

[35] Ko H, Hofer SB, Pichler B, Buchanan KA, Sjöström PJ, et al (2011) Functional specificity of local synaptic connections in neocortical networks. Nature 473: 87–91. 21478872 10.1038/nature09880 PMC3089591

[36] Ko H, Cossell L, Baragli C, Antolik J, Clopath C, et al (2013) The emergence of functional microcircuits in visual cortex. Nature 496: 96–100. 23552948 10.1038/nature12015 PMC4843961

[37] Yoshimura Y, Callaway EM (2005) Fine-scale specificity of cortical networks depends on inhibitory cell type and connectivity. Nat Neurosci 8: 1552–1559. 16222228 10.1038/nn1565

[38] Hofer SB, Ko H, Pichler B, Vogelstein J, Ros H, et al (2011) Differential connectivity and response dynamics of excitatory and inhibitory neurons in visual cortex. Nat Neurosci 14: 1045–1052. 21765421 10.1038/nn.2876 PMC6370002

[39] Gabbott PL, Somogyi P (1986) Quantitative distribution of GABA-immunoreactive neurons in the visual cortex (area 17) of the cat. Exp Brain Res 61: 323–331. 3005016 10.1007/BF00239522

[40] Beaulieu C (1993) Numerical data on neocortical neurons in adult rat, with special reference to the GABA population. Brain Res 609: 284–292. 8508310 10.1016/0006-8993(93)90884-p

[41] Douglas RJ, Koch C, Mahowald M, Martin KA, Suarez HH (1995) Recurrent excitation in neocortical circuits. Science 269: 981–985. 7638624 10.1126/science.7638624

[42] Stepanyants A, Hirsch JA, Martinez LM, Kisvárday ZF, Ferecskó AS, et al (2008) Local potential connectivity in cat primary visual cortex. Cerebral Cortex 18: 13–28. 17420172 10.1093/cercor/bhm027

[43] Hubel D, Wiesel T (1962) Receptive fields, binocular interaction and functional architecture in the cat's visual cortex. J Physiol 160: 106–154. 14449617 10.1113/jphysiol.1962.sp006837 PMC1359523

[44] Coleman JE, Law K, Bear MF (2009) Anatomical origins of ocular dominance in mouse primary visual cortex. Neuroscience 161: 561–571. 19327388 10.1016/j.neuroscience.2009.03.045 PMC2735235

[45] Stosiek C, Garaschuk O, Holthoff K, Konnerth A (2003) In vivo two-photon calcium imaging of neuronal networks. Proceedings of the National Academy of Sciences of the United States of America 100: 7319–7324. 12777621 10.1073/pnas.1232232100 PMC165873

[46] Nimmerjahn A, Kirchhoff F, Kerr JND, Helmchen F (2004) Sulforhodamine 101 as a specific marker of astroglia in the neocortex in vivo. Nat Methods 1: 31–37. 15782150 10.1038/nmeth706

[47] Straw AD (2008) Vision egg: an open-source library for realtime visual stimulus generation. Front Neuroinform 2: 4. 19050754 10.3389/neuro.11.004.2008 PMC2584775

[48] Ringach DL, Shapley RM, Hawken MJ (2002) Orientation selectivity in macaque V1: diversity and laminar dependence. J Neurosci 22: 5639–5651. 12097515 10.1523/JNEUROSCI.22-13-05639.2002 PMC6758222

[49] Niell CM, Stryker MP (2008) Highly selective receptive fields in mouse visual cortex. J Neurosci 28: 7520–7536. 18650330 10.1523/JNEUROSCI.0623-08.2008 PMC3040721

[50] Nevian T, Helmchen F (2007) Calcium indicator loading of neurons using single-cell electroporation. Pflugers Arch - Eur J Physiol 454: 675–688. 17334778 10.1007/s00424-007-0234-2

[51] Cardona A, Saalfeld S, Schindelin J, Arganda-Carreras I, Preibisch S, et al (2012) TrakEM2 Software for Neural Circuit Reconstruction. PLoS ONE 7: e38011. 22723842 10.1371/journal.pone.0038011 PMC3378562

[52] Gray E (1959) Axo-somatic and axo-dendritic synapses of the cerebral cortex: an electron microscope study. J Anat 93: 420–433. 13829103 PMC1244535

[53] Colonnier M (1968) Synaptic patterns on different cell types in the different laminae of the cat visual cortex. An electron microscope study. Brain Res 9: 268–287. 4175993 10.1016/0006-8993(68)90234-5

[54] Peters A, Palay S, Webster F (1991) The fine structure of the nervous system: neurons and their supporting cells. 3rd ed. New York: Oxford University Press.

[55] Gundersen H, Jensen E (1987) The efficiency of systematic sampling in stereology and its prediction. J Microsc 147: 229–263. 3430576 10.1111/j.1365-2818.1987.tb02837.x

[56] Saalfeld S, Cardona A, Hartenstein V, Tomancak P (2009) CATMAID: collaborative annotation toolkit for massive amounts of image data. Bioinformatics 25: 1984–1986. 19376822 10.1093/bioinformatics/btp266 PMC2712332

