# Supplementary figures and images for: Pyramidal Cells Make Specific Connections onto Smooth (GABAergic) Neurons in Mouse Visual Cortex
Source: PLoS Biol. 2014 Aug 19;12(8):e1001932. doi: 10.1371/journal.pbio.1001932 (PMC4138028; doi:10.1371/journal.pbio.1001932)

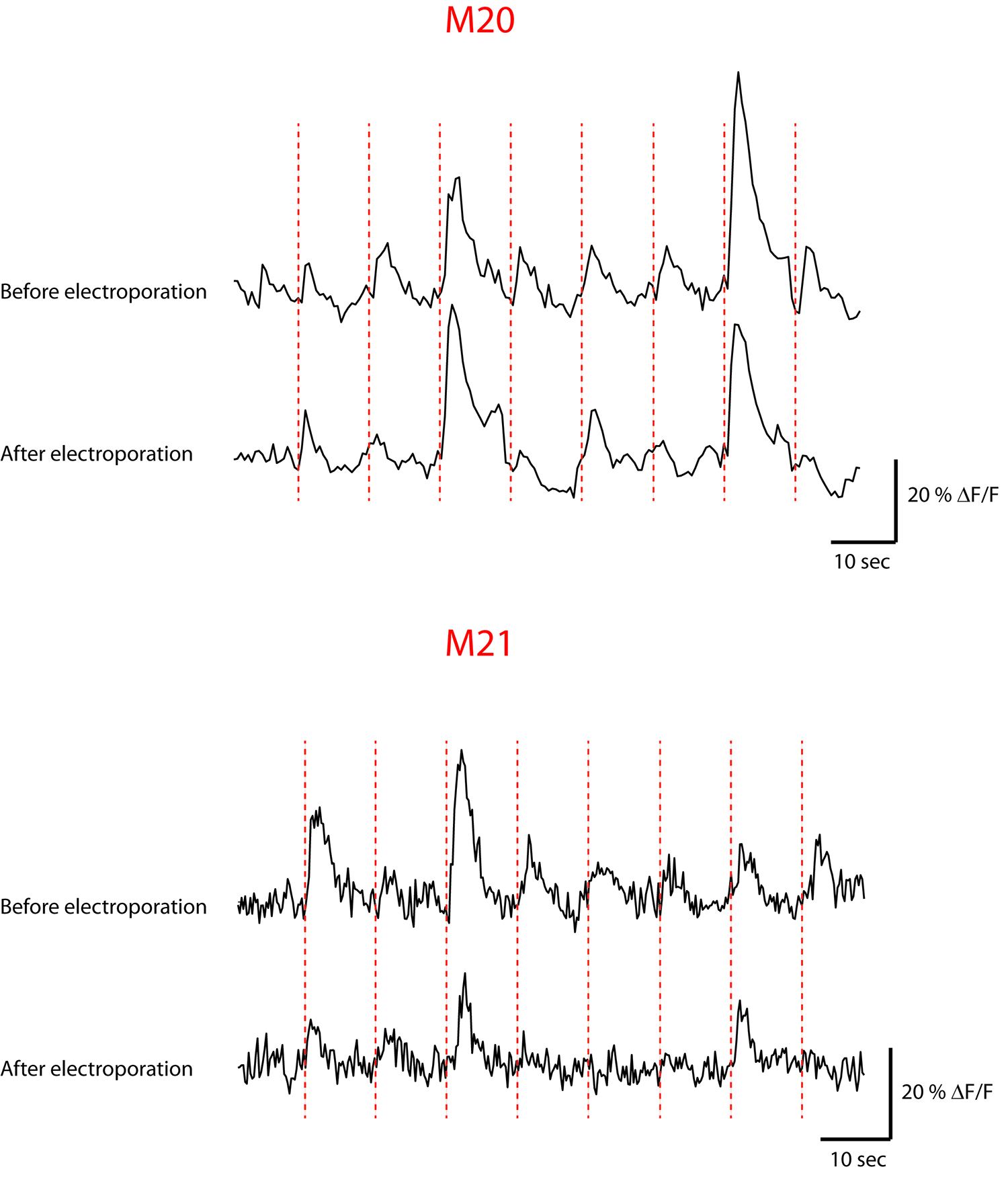

Supplement: Figure S1 — Examples of responses before and after electroporation for the cells M20 and M21. Black traces are the averaged responses to drifting gratings. Stimulation onsets are indicated by orange dotted lines. (TIF) [file pbio.1001932.s001.tif]
